# Comparative analysis of five obesity-related indicators for predicting infertility in U.S. adults

**DOI:** 10.3389/fnut.2025.1593706

**Published:** 2025-06-24

**Authors:** Ke-Qin Yu, Wei-Qiang Zhao, Ting Zhang

**Affiliations:** 1 Department of Gynaecology & Obstetrics, The First Affiliated Hospital of Zhejiang Chinese Medical University (Zhejiang Provincial Hospital of Chinese Medicine), Hangzhou, China; 2 The First Clinical College of Zhejiang Chinese Medical University, Hangzhou, China; 3 Department of Orthopaedic & Traumatology, The First Affiliated Hospital of Zhejiang Chinese Medical University (Zhejiang Provincial Hospital of Chinese Medicine), Hangzhou, China

**Keywords:** body roundness index, obesity-index, relative fat mass, lipid accumulation product, National Health and Nutrition Examination Survey

## Abstract

**Objective:**

Infertility is increasingly prevalent worldwide, emerging as a significant endocrine disorder of global concern. This study sought to explore associations between infertility and five distinct obesity-related metrics: body roundness index (BRI), relative fat mass (RFM), body mass index (BMI), lipid accumulation product (LAP), and waist circumference (WC). Evaluated and compared the predictive performance of these indicators in screening for infertility additionally.

**Methods:**

This research utilized data from the 2013–2018 cycles of the National Health and Nutrition Examination Survey (NHANES). Weighted logistic regression analyses with multi-model adjustments were performed to examine the relationship between five specific indicators and infertility. The diagnostic potential of five indicators was evaluated through receiver operating characteristics curve (ROC). Two part linear regression models are also used to estimate threshold effects. The association between the indicators and infertility was examined using smooth curve fitting techniques, while subgroup analyses were conducted to identify variations in risk across different population segments.

**Results:**

The study included 3,528 participants from NHANES 2013–2018, comprising 365 individuals with infertility and 3,163 without. Weighted multivariate logistic regression analysis identified BRI, RFM, BMI, WC, and LAP as significant predictors of infertility. The odds ratios for the highest quartiles were 2.56 for BRI, 2.45 for RFM, 2.38 for BMI, 2.33 for WC, and 1.40 for LAP. Optimal thresholds were determined as 6.47 for BRI, 36.4 for BMI, 30.29 for RFM, 119.20 for WC, and 19.15 for LAP. The area under the ROC curve for BRI was 0.651, indicating moderate predictive performance. Subgroup analyses revealed that individuals aged over 35, smokers, and those with diabetes or hypertension were more likely to report infertility.

**Conclusion:**

All five obesity-related indicators were positively associated with infertility in the U.S. population. Among them, BRI demonstrated relatively stronger predictive performance. Beyond the natural influence of aging, particular attention should be directed toward the prevention of smoking, diabetes, and hypertension to mitigate associated risks.

## 1 Introduction

The failure to conceive after more than a year of consistent, unprotected sexual activity despite having normal sexual activity is known as infertility. With research showing that the frequency of infertility among people of reproductive age ranges from 10% to 18% and is rapidly rising, this global health concern has gained increasing attention (1). In addition to impairing people’s and families’ ability to procreate, infertility places a heavy psychological and financial strain on those affected (2, 3). Every year, about 12.7% of American women who are of reproductive age seek therapy for infertility (4).

Obesity, recognized as a modern epidemic, has attracted increasing research attention regarding its impact on infertility (5). The rising prevalence of obesity among women of childbearing age has been identified as a key factor contributing to infertility (6, 7). Body mass index (BMI) is a widely recognized and utilized metric for assessing obesity, commonly applied in health evaluations and clinical research. However, it is important to acknowledge that conventional measures like BMI and waist circumference (WC) have limitations in accurately capturing critical aspects of body composition, including the distribution of adipose tissue, visceral fat accumulation, and the metabolic regulation of lipids. In recent years, innovative assessment methods have emerged that integrate BMI, WC, and metabolic markers such as lipid. These tools aim to provide a more comprehensive evaluation of obesity-related features by incorporating multiple physiological dimensions. Relative fat mass (RFM) serves as an alternative body composition metric that quantifies the proportion of total body fat. Unlike BMI, RFM offers a more precise assessment of fat distribution across the body (8). The Lipid Accumulation Product (LAP) is more precise metrics for assessing body fat distribution and visceral fat accumulation (9). The body roundness index (BRI), based on an elliptical model of the human body and the concept of eccentricity, provides an effective measure of visceral fat accumulation by incorporating WC and height (Ht) (10, 11). Beyond reflecting visceral and overall body fat distribution, BRI has been associated with a range of health conditions, including metabolic syndrome, digestive system disorders, and mental health issues (12–16).

Despite numerous studies examining the association between anthropometric indices (BMI, RFM, LAP) and infertility (17, 18), identifying the most effective predictor remains challenging. In this study, we compared a newly developed screening tool with traditional indicators, comprehensively evaluated the predictive potential of nine obesity-related measures for infertility risk, and aimed to identify the method with the strongest predictive capacity. Our findings seek to provide new insights into the prediction and clinical management of infertility.

## 2 Methods

### 2.1 Study design and population

The data for this study were sourced from the U.S. National Health and Nutrition Examination Survey (NHANES), a CDC-led program evaluating the health and nutritional status of the U.S. population using a multi-stage probability sampling method (19, 20). Conducted from 2013 to 2018, NHANES collects data through household interviews, health screenings, and lab tests, with ethical approval from the National Center for Health Statistics (NCHS) and informed consent from all participants. Additional details are available on the NHANES website (https://www.cdc.gov/nchs/nhanes/index.htm).

**Figure 1:**
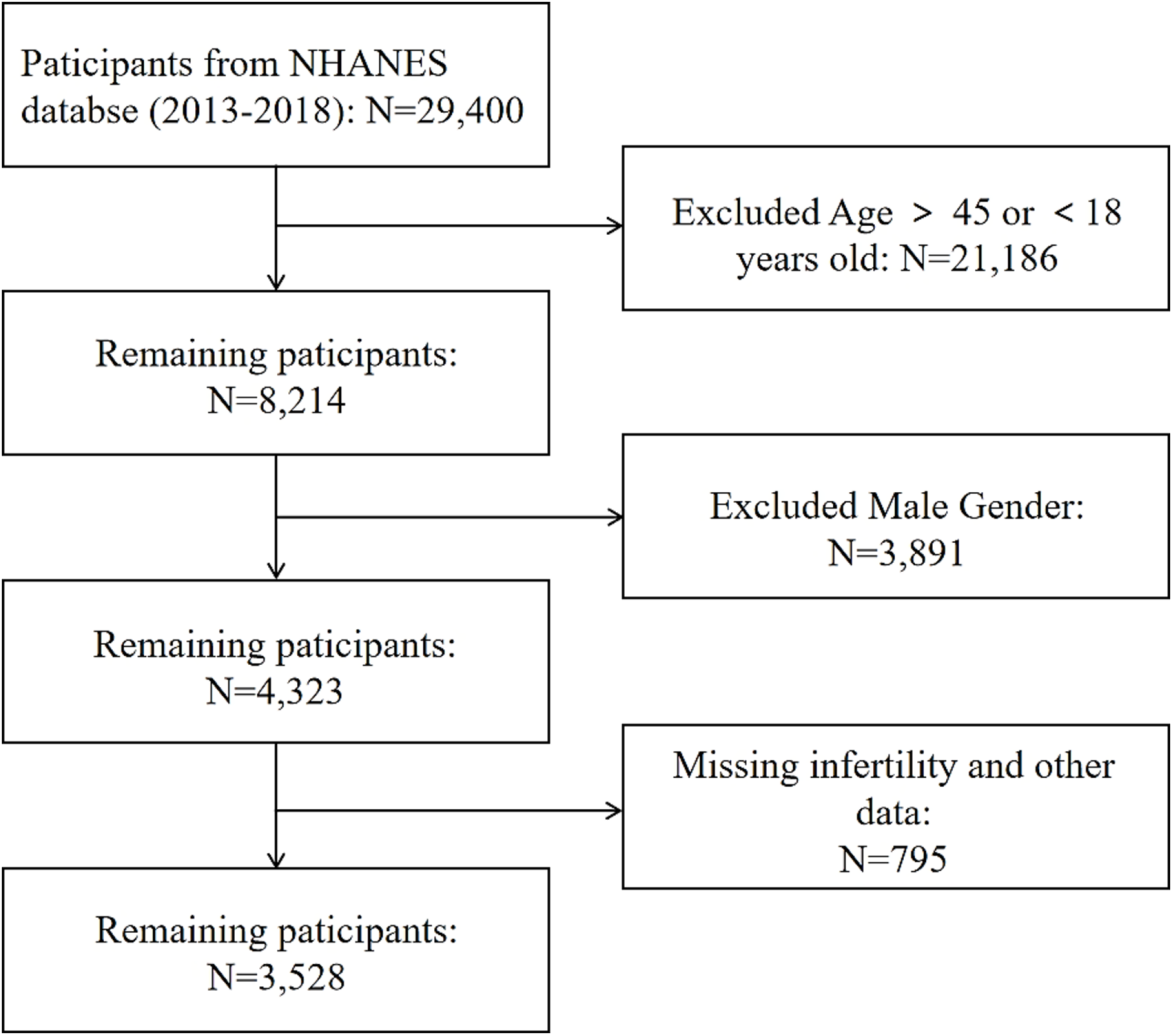
The flowchart of participants selection data from NHANES 2013 to 2018 for American adults.

A total of 29,400 participants were enlisted initially for this analysis. Our final analysis comprised 3,528 eligible participants (Figure 1) after removing improper age (*N* = 21,186), male participants (*N* = 3,891), lacking infertility data and lacking information examination data on five body measurements (BMI, WC, LAP, BRI and RFM) (*N* = 795).

### 2.2 Data collection

According to the NHANES database website, the database encompasses demographic data such as race, gender, and age; anthropometric measurements including WC, Ht, WC and BMI; laboratory data such as triglycerides, total cholesterol (TC), high-density lipoprotein (HDL-C), and low-density lipoprotein (LDL-C); as well as questionnaire data covering reproductive health, alcohol consumption habits, smoking habits, the poverty-income ratio (PIR), medical conditions, and more. A team of trained researchers collected basic information about the participants. Regularly trained health technicians gathered blood and urine samples along with anthropometric data at Mobile Examination Centers (MECs), which were then sent to designated examination centers for standardized testing.

### 2.3 Definition of infertility

In the Questionnaire Data, each female participant’s self-report was used as the dependent variable for infertility (questionnaire variable name: RHQ074). Researchers utilized questions such as “Have you been trying to conceive for one year?” to assess the subjects. A response of “Yes” was categorized as “infertility,” while a response of “No” was categorized as “non-infertility.”

### 2.4 Definition of five obesity-related screening indicators

**Figure 2:**
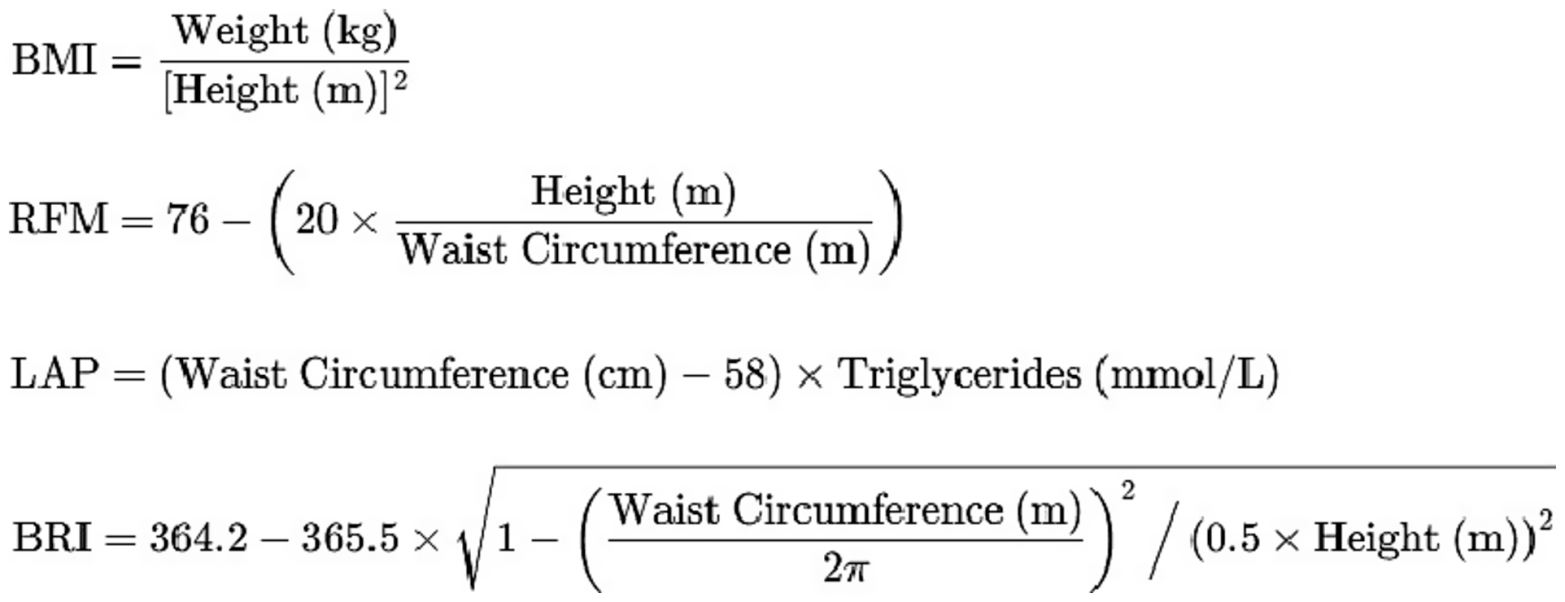
Calculation of five obesity-related indicators.

In this study, the five screening measures associated with obesity include BMI, WC, LAP, BRI, RFM. WC and Ht were measured by health technicians trained in anthropometric assessments at the MECs. Ht was measured barefoot using a stadiometer, ensuring head alignment. WC was recorded at the end of exhalation, positioned just above the iliac crest along the mid-axillary line. HDL-C, triglycerides data, which are the other indicators required in the formula, were analyzed by the University of Minnesota laboratory under strict surveillance and quality management. The calculation of five obesity-related indicators were showed in Figure 2.

### 2.5 Covariables

Our study also considered additional factors, including age (years old), PIR, race and ethnicity (Mexican American/Other Hispanic/Non-Hispanic White/Non-Hispanic Black/Other race), education level (<9th grade, 9–11th grade, high school graduate, some college or AA degree, and college graduate or above), marital status (married, widowed, divorced, separated, never married and living with partner), BMI (kg/m^2^), Ht (m), weight (Wt, kg), WC (cm or m), triglycerides (mmol/L), TC (mmol/L), HDL-C (mmol/L), LDL-C (mmol/L), diabetes (yes/no), hypertension (yes/no), smoking at least 100 cigarettes (yes/no), and consuming at least 12 alcoholic drinks per year (yes/no). These variables may influence the relationship between screening indicators and infertility. The complete measurement procedures for these variables are publicly available at www.cdc.gov/nchs/nhanes/.

### 2.6 Statistical analysis

In accordance with the analytic guidelines of the Centers for Disease Control and Prevention (CDC), all analyses accounted for the complex, multistage probability sampling design of NHANES, including sampling weights, strata, and primary sampling units (PSUs). Survey design variables, stratum (SDMVSTRA), PSU (SDMVPSU), and examination weights (WTMEC2YR).

In descriptive analyses, a weighted Student’s *t*-test (for continuous variables) or a weighted Chi-square test (for categorical data) were used to evaluate the two comparison groups based on their infertile status: infertility and non-infertility. Classification parameters are expressed as a ratio, whereas the mean plus standard deviation summarizes continuous data. The distribution of continuous variables was assessed using Shapiro–Wilk test. Variables that followed a normal distribution were reported as mean ± standard deviation (SD), while non-normally distributed variables were presented as median and interquartile range (IQR). Investigating the relationship between five screening indicators and infertility included using a multivariate regression model that took into account the NHANES complicated sampling design (sampling weights). Covariates in Model 1 were not altered in any way. Age, educational level, and race were modified in Model 2. Model 3 took into account the following variables: age, education level, PIR, marry status, smoking status, alcohol use status, diabetes and hypertension status. Subgroup analysis was conducted using a hierarchical multivariable logistic regression model with stratified covariates, including age (<35 years old and >35 years old), race and ethnicity (Mexican American, other Hispanic, non-Hispanic White, non-Hispanic Black and other race), education level (<9th grade, 9–11th grade, high school graduate, some college or AA degree, and college graduate or above), marital status (married, widowed, divorced, separated, never married and living with partner), PIR, smoking at least 100 cigarettes (yes/no), drinking at least 12 times/year (yes/no), diabetes status (yes/no/border) and hypertension status (yes/no).

To assess the nonlinear association between the five screening indicators and infertility, this research used a weighted generalized additive model regression and smoothed curve fitting. Use a two-stage linear regression model (segmented regression model) to fit each period and calculate the threshold effect when presenting non-linear connections. If non-linear correlations are found, the threshold effect is determined by fitting each interval using a two-stage linear regression model. Apply a two-step recursive technique to find the turning point (K) of the connecting line segment, which is based on the model that generates the maximum likelihood. We also plotted receiver operating characteristic curve (ROC) and computed the area under the curve (AUC) values to evaluate the predictive power of the different indicators. Furthermore, bootstrap resampling (carried out 500 times) was conducted as a sensitivity analysis to assess the stability of AUC. All analyses were conducted using R 4.1.2 version (R Foundation for Statistical Computing, Vienna, Austria) and Empower software (www.Empowerstats.com; X&Y solutions, Inc., Boston, Massachusetts). The ‘survey’ package is used for analysis of complex sample designs, ‘mgcv’ for generalized additive models and smooth curve fitting, and ‘pROC’ for ROC curve analysis and AUC calculation. *p* < 0.05 is considered a statistically significant level.

## 3 Results

### 3.1 Baseline characteristics of study participants

A total of 3,528 participants were enrolled from 2013 to 2018, of which 365 were infertility, and 3,163 participants were non-infertility, with a prevalence of 10.34%. Based on their infertility status, the characteristics of the study participants are shown in Table 1. Self-reported infertility is more prevalent among women who are older, have higher body weight, larger waist circumference, elevated BMI, and engage in drinking. Significant differences were observed between self-reported infertility and non-infertility patients in demographic factors (race, ethnicity, age, PIR, marital status), health conditions (diabetes, hypertension, smoking, drinking), and clinical measures (Ht, Wt, HDL-C, LDL-C, TC). Additionally, five screening indicators—WC, BMI, BRI, LAP and RFM, also showed significant difference.

**Table 1:** The baseline of characteristics of study population from NHANES 2013–2018.

Variables	Infertility	Non-infertility	*p*-value
Number	*N* = 365	*N* = 3,163	
Age, years (mean±SD)	34.79 ± 7.14	30.76 ± 8.43	<0.001
<35 years old, *n* (%)	161 (44.11)	2002 (63.29)	
≥35 years old	204 (55.89)	1,161 (36.71)	
Height, m	1.62 ± 0.07	1.61 ± 0.07	0.031
BMI (kg/m^2^)	31.00 [24.70–37.30]	27.60 [22.88–33.80]	<0.001
Weight, kg	80.40 [64.70–100.90]	71.50 [75.80–77.31]	<0.001
Waist circumference, cm	100.00 [63.40–169.60]	92.00 [80.70–106.20]	<0.001
Triglyceride, mmol/L	1.14 [0.60–3.96]	1.25 [0.54–4.02]	0.358
HDL-C, mmol/L	1.34 [1.09–1.55]	1.40 [1.16–1.68]	<0.001
LDL-C, mmol/L	2.43 [1.53–3.15]	2.20 [0.98–2.92]	<0.001
Total cholesterol, mmol/L	4.71 [4.16–5.33]	4.53 [3.98–5.17]	0.002
PIR	2.67 ± 1.64	2.39 ± 1.64	<0.001
Classification, *n* (%)			0.115
≤1.3	120 (32.88)	1,202 (38.00)	
>1.3 and ≤3.5	139 (38.08)	1,164 (36.80)	
>3.5	106 (29.04)	797 (25.20)	
Race and ethnicity, *n* (%)			0.037
Mexican American	61 (16.71)	556 (17.58)	
Other Hispanic	30 (8.22)	339 (10.72)	
Non-Hispanic White	142 (38.90)	1,006 (31.81)	
Non-Hispanic Black	75 (20.55)	702 (22.19)	
Other race	57 (15.62)	560 (17.70)	
Education level, *n* (%)			0.815
<9th grade	14 (3.84)	161 (5.09)	
9–11th grade	38 (10.41)	343 (10.84)	
High school graduate	71 (19.45)	628 (19.85)	
Some college or AA degree	144 (39.45)	1,177 (37.21)	
College graduate or above	98 (26.85)	854 (27.00)	
Marital status, *n* (%)			<0.001
Married	219 (60.00)	1,214 (38.38)	
Widowed	3 (0.82)	14 (0.44)	
Divorced	27 (7.40)	199 (6.29)	
Separated	15 (4.11)	121 (3.83)	
Never married	55 (15.07)	1,115 (35.25)	
Living with partner	46 (12.60)	500 (15.81)	
Alcohol use, *n* (%)			0.033
Yes	273 (724.79)	2,195 (69.40)	
No	92 (25.21)	968 (30.60)	
Smoking, *n* (%)			0.010
Yes	131 (35.89)	814 (25.74)	
No	234 (64.11)	2,349 (74.26)	
Diabetes, *n* (%)			0.003
Yes	30 (8.22)	112 (3.54)	
No	328 (89.86)	3,006 (95.04)	
Border	7 (1.92)	45 (1.42)	
Hypertension, *n* (%)			<0.001
Yes	75 (20.55)	415 (13.12)	
No	290 (79.45)	2,748 (86.88)	
BRI	6.44 ± 3.02	5.31 ± 2.67	<0.001
RFM	43.18 ± 6.18	40.53 ± 6.42	<0.001
LAP	77.58 ± 86.89	64.82 ± 86.96	<0.001

SD, standard deviation; BMI, body mass index; PIR, poverty-to-income ratio; HDL-C, high density lipoprotein; LDL-C, low density lipoprotein; BRI, body roundness index; RFM: relative fat mass; LAP, lipid accumulation product.

Data are presented as median [interquartile range, IQR] for non-normally distributed variables, and as mean ± SD for normally distributed variables.

### 3.2 The association between BRI and infertility

The association between five screening indicators and infertility is seen in Table 2, and the indicators were analyzed as continuous variables, quartiles, and their trend. After adjusting for all relevant covariates, all positive relationships persist in Model 3 from five screening indicators, where the highest odds ratio (OR) of BRI is 1.120 (95% CI: 1.076–1.165). And the followed was RFM (OR = 1.062, 95% CI: 1.041–1.084), BMI (OR = 1.034, 95% CI: 1.020–1.048), WC (OR = 1.019, 95% CI: 1.013–1.026) and LAP (OR = 1.002, 95% CI: 1.000–1.003).

**Table 2:** Relationship between five obesity-related index and infertility and non-infertility in different models.

Variables	OR (95% CI), *p*-value		
	Model 1	Model 2	Model 3
**BRI**	1.138 (1.099–1.178), <0.001	1.137 (1.095–1.180), <0.001	1.120 (1.076–1.165), <0.001
Quartile of BRI			
Q1 (<3.4)	Reference	Reference	Reference
Q2 (3.4–4.9)	1.443 (0.998–2.086), 0.051	1.299 (0.893–1.889), 0.171	1.292 (0.924–1.624), 0.141
Q3 (4.9–6.9)	2.000 (1.409–2.839), <0.001	1.859 (1.296–2.666),<0.001	1.791 (1.244–2.578), 0.002
Q4 (≥6.9)	2.991 (2.142–4.177), <0.001	2.857 (2.020–4.039), <0.001	2.561 (1.792–3.662), <0.001
*p* for trend	<0.001	<0.001	<0.001
**WC**	1.022 (1.016–1.028), <0.001	1.021 (1.015–1.028), <0.001	1.019 (1.013–1.026), <0.001
Quartile of WC			
Q1 (<81.2)	Reference	Reference	Reference
Q2 (81.2–92.3)	1.284 (0.892–1.847), 0.178	1.192 (0.824–1.727), 0.351	1.173 (0.887–1.542), 0.237
Q3 (92.3–106.4)	1.708 (1.208–2.416), 0.002	1.602 (1.120–2.292), 0.009	1.540 (1.072–2.212), 0.002
Q4 (≥106.4)	2.829 (2.045–3.916), <0.001	2.623 (1.863–3.693), <0.001	2.334 (1.637–3.329), <0.001
*p* for trend	<0.001	<0.001	<0.001
**RFM**	1.071 (1.052–1.090), <0.001	1.070 (1.049–1.091), <0.001	1.062 (1.041–1.084), <0.001
Quartile of RFM			
Q1 (<36.2)	Reference	Reference	Reference
Q2 (36.2–41.2)	1.442 (0.998–2.086), 0.178	1.203 (0.801–1.452), 0.351	1.252 (0.779–1.701), 0.332
Q3 (41.2–45.9)	1.956 (1.368–2.339), <0.001	1.878 (1.316–2.662), <0.001	1.774 (1.156–2.234), 0.002
Q4 (≥45.9)	2.994 (2.156–4.025), <0.001	2.721 (2.165–4.091), <0.001	2.451 (1.717–3.024),<0.001
*p* for trend	<0.001	<0.001	<0.001
**LAP**	1.002 (1.000–1.003), 0.003	1.002 (1.000–1.003), 0.016	1.002 (1.000–1.003), 0.026
Quartile of LAP			
Q1 (<11.9)	Reference	Reference	Reference
Q2 (11.9–34.1)	1.350 (0.968–1.884), 0.077	1.310 (0.935–1.835), 0.116	1.228 (0.874–1.727), 0.236
Q3 (34.1–91.6)	1.647 (1.194–2.273), 0.002	1.603 (1.157–2.222), 0.005	1.567 (1.128–2.177), 0.007
Q4 (≥91.6)	1.592 (1.512–2.200), 0.005	1.476 (1.061–2.053), 0.021	1.402 (1.005–1.956),0.046
*p* for trend	<0.001	<0.001	0.024
**BMI**	1.039 (1.027–1.052), <0.001	1.040 (1.026–1.053), <0.001	1.034 (1.020–1.048), <0.001
Quartile of BMI			
Q1 (<23)	Reference	Reference	Reference
Q2 (23–27.8)	1.173 (0.817–1.685), 0.388	1.093 (0.757–1.577), 0.636	1.088 (0.994–1.573), 0.053
Q3 (27.8–33.9)	1.643 (1.169–2.310), 0.004	1.596 (1.124–2.265), 0.009	1.531 (1.075–2.180), 0.018
Q4 (≥33.9)	2.673 (1.942–3.681), <0.001	2.658 (1.907–3.704), <0.001	2.375 (1.687–3.343), <0.001
*p* for trend	<0.001	<0.001	<0.001

Model 1: adjusted for no covariates.

Model 2: adjusted for education level, age, race.

Model 3: adjusted for education level, age, race, PIR, martial status, smoking status, alcohol use status, diabetes and hypertension status.

BRI, body roundness index; BMI, body mass index; WC, waist circumference; RFM, relative fat mass; LAP, lipid accumulation product; CI, confidence interval; OR, odds ratio; PIR, poverty to income ratio.

In the highest quartile (Q4), BRI showed the strongest association and relatively higher predictive value among the five indicators evaluated (OR = 2.561, 95% CI: 1.792–3.662), followed by RFM (OR = 2.451, 95% CI: 1.717–3.024), BMI (OR = 2.375, 95% CI: 1.687–3.343), WC (OR = 2.334, 95% CI: 1.637–3.329), and LAP (OR = 1.402, 95% CI: 1.005–1.956).

### 3.3 Subgroup analysis

**Figure 3:**
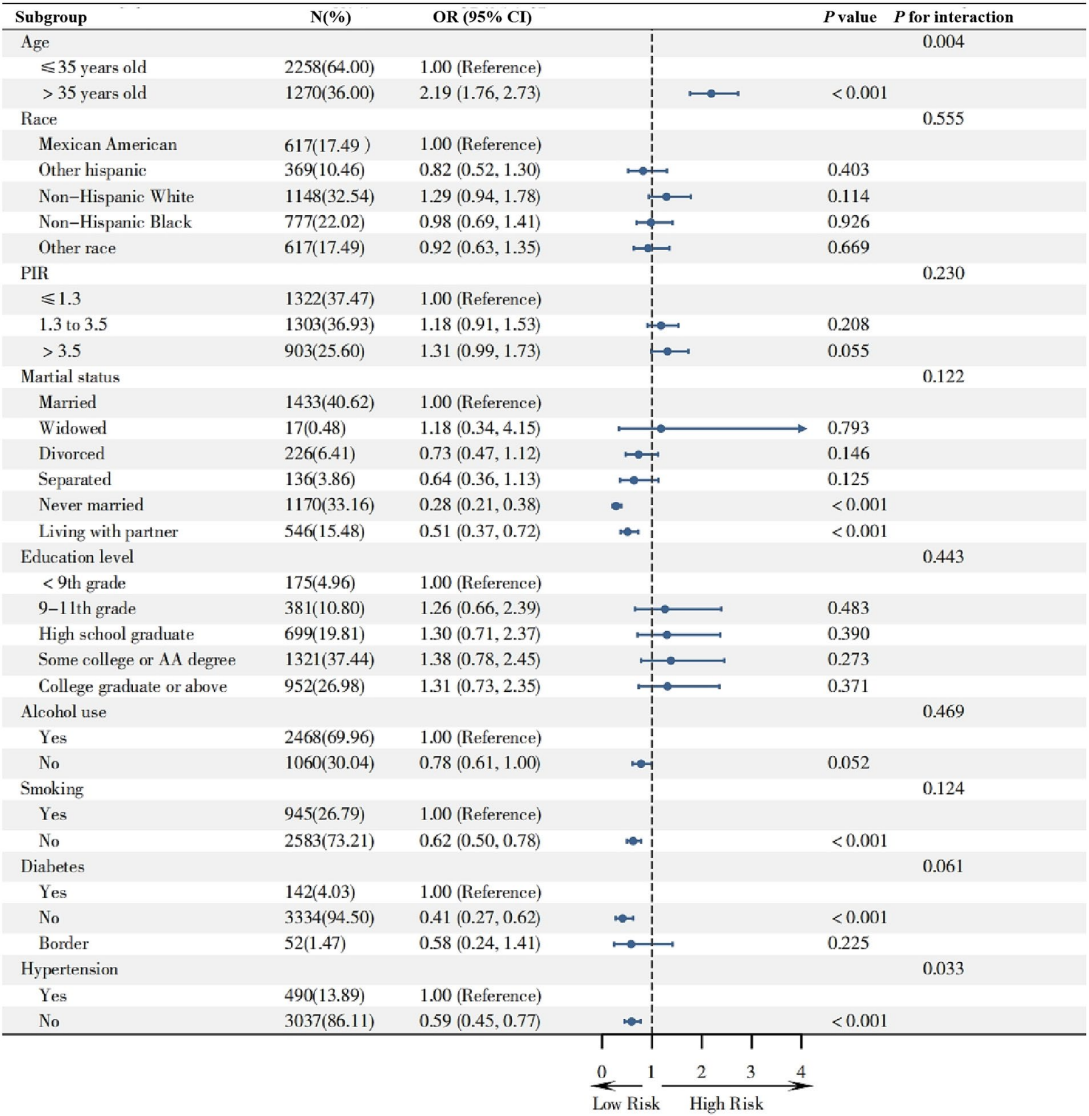
The forest plot analysis of the association between different subgroups and infertility.

We conducted subgroup analyses (Figure 3) to evaluate the stability of the relationship between the five screening indicators and infertility across various demographic and health conditions. Participants aged over 35 years had a significantly increased risk of infertility (OR = 2.19, 95% CI: 1.76–2.73). Compared to married individuals, those who were never married or living with a partner showed protective effects against infertility. Additionally, non-smokers and participants without diabetes or hypertension exhibited a reduced risk of infertility. Significant interaction effects were observed for age group and self-reported hypertension (*p* < 0.05 for both).

### 3.4 Non-linearity and saturation effect analysis between indicators and infertility

**Figure 4:**
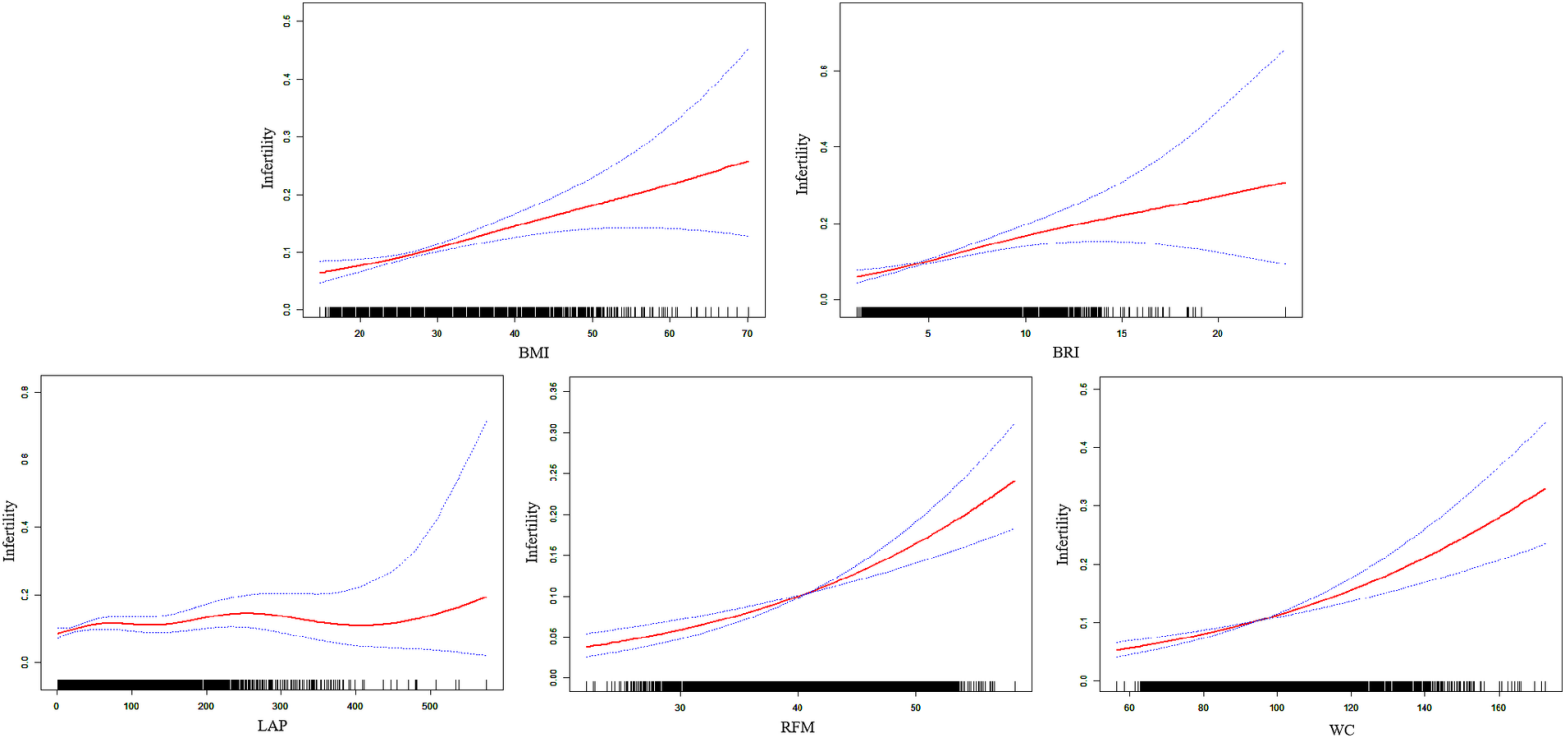
The non-linear relationship between five obesity-indicators and infertility.

The nonlinear relationships between the screening indicators and infertility were examined using weighted generalized additive models and smooth curve fitting. The results were presented in Figure 4, illustrate the patterns of association for all five indicators. And Table 3 showed threshold effect analysis of five screening indicators on infertility risk.

**Table 3:** The threshold effect analysis of five screening indicators on infertility risk among adults in NHANES 2013–2018.

Variables	OR (95% CI)	*p*-value
BRI		
Model 1		
The standard linear model	1.14 (1.10–1.17)	<0.001
Model 2		
Turning point (K)	6.47	
BRI < 6.47	1.27 (1.13–1.43)	<0.001
BRI ≥ 6.47	1.05 (0.98–1.13)	0.149
Log likelihood ratio test		0.013
WC		
Model 1		
The standard linear model	1.02 (1.01–1.03)	<0.001
Model 2		
Turning point (K)	119.20	
BRI < 119.20	1.02 (1.01–1.03)	<0.001
BRI ≥ 119.20	1.01 (0.98–1.03)	0.564
Log likelihood ratio test		0.134
RFM		
Model 1		
The standard linear model	1.07 (1.05–1.09)	<0.001
Model 2		
Turning point (K)	30.29	
BRI < 30.29	0.97 (0.76–1.24)	0.808
BRI ≥ 30.29	1.07 (1.05–1.09)	<0.001
Log likelihood ratio test		0.459
BMI		
Model 1		
The standard linear model	1.04 (1.02–1.05)	<0.001
Model 2		
Turning point (K)	36.40	
BMI < 36.40	1.06 (1.04–1.08)	<0.001
BMI ≥ 36.40	1.01 (0.98–1.05)	0.352
Log likelihood ratio test		0.042
LAP		
Model 1		
The standard linear model	1.00 (1.00–1.00)	0.001
Model 2		
Turning point (K)	19.15	
BMI < 19.15	1.03 (1.01–1.06)	0.003
BMI ≥ 19.15	1.00 (0.99–1.00)	0.260
Log likelihood ratio test		0.004

Model 1 was a standard linear model, and model 2 was a piecewise model. Log likelihood ratio test was used to compare two models.

Abbreviations: BRI, body roundness index; WC, waist circumference; RFM, relative fat mass; BMI, body mass index; LAP, lipid accumulation product.

### 3.5 Indicators for predicting infertility

**Figure 5:**
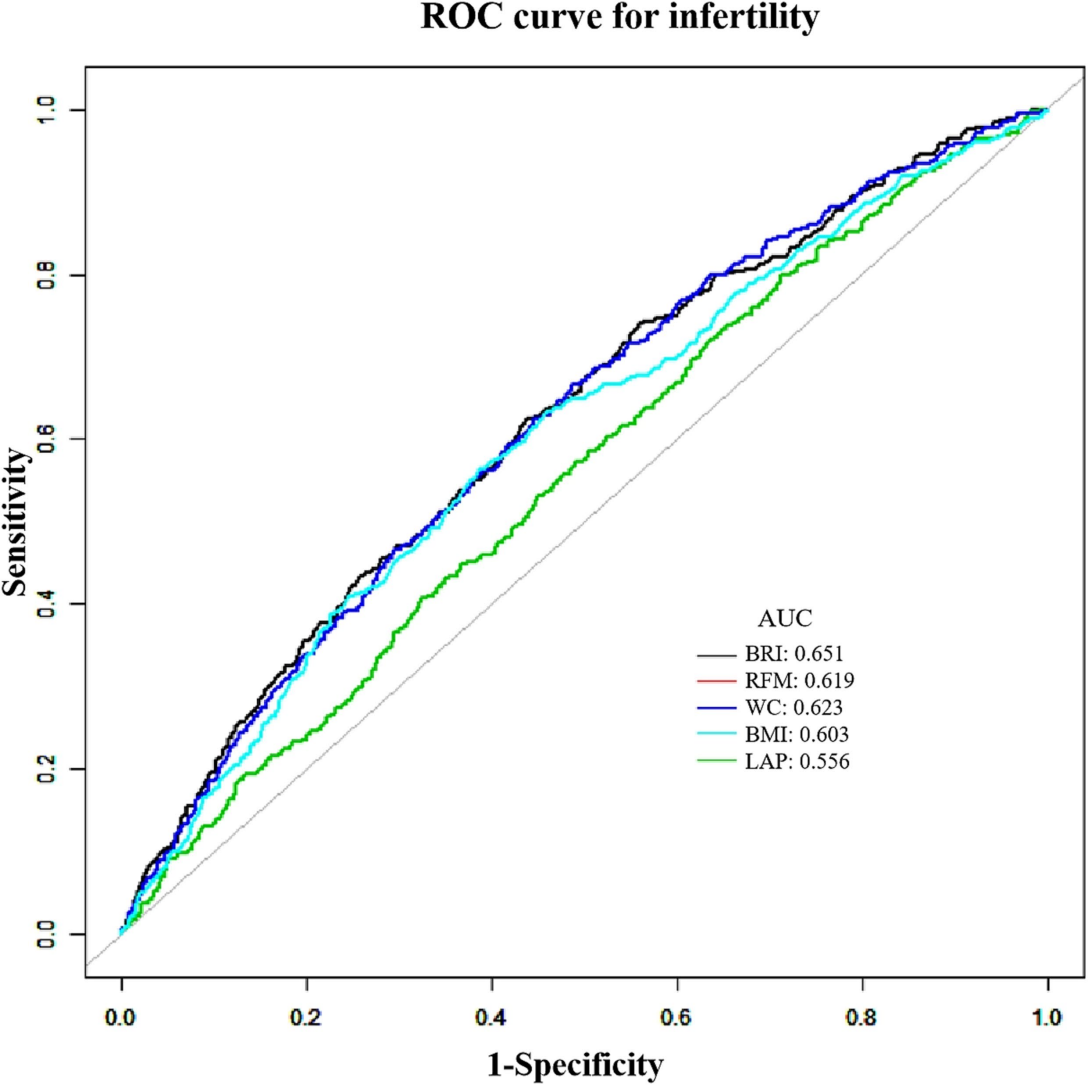
ROC curves for five screening indicators for predicting infertility.

Figure 5 and Table 4 provided the AUC levels and 95% CI for the five indices screening for infertility in US adults. BRI was the most discriminating indicator for infertility (AUC = 0.651), followed by WC (AUC = 0.623). In contrast, LAP yielded a lower AUC of 0.556. However, all indices demonstrated AUC values above 0.5, indicating their statistically meaningful diagnostic capability for infertility detection.

**Table 4:** Results of ROC analysis of five screening index.

Variables	AUC	95% CI lower	95% CI upper	Specificity	Sensitivity
BRI	0.651	0.613	0.682	0.637	0.711
RFM	0.619	0.587	0.649	0.594	0.607
WC	0.623	0.593	0.653	0.562	0.624
BMI	0.603	0.571	0.634	0.613	0.564
LAP	0.556	0.525	0.586	0.288	0.798

Abbreviations: BRI, body roundness index; BMI, body mass index; WC, waist circumference; RFM, relative fat mass; LAP, lipid accumulation product; CI, confidence interval.

## 4 Discussion

To our knowledge, this represents the first comprehensive investigation into the association between five widely used obesity-related anthropometric measures and infertility within the US population. This study investigated the relationship between infertility and the indicies in a cohort of 3,528 American women from the NHANES 2013–2018. Through the cycles, we found the prevalence of infertility in the U.S. was 10.34%. After fully adjusting for potential confounders, including age, education level, marital status, smoking, alcohol consumption, hypertension, diabetes, PIR, the association remained statistically significant.

Global population surveys show that infertility has been a major focus in the field of reproductive health for many years (5). Infertility not only elevates the risk of developing cancers such as breast cancer, endometrial cancer, and ovarian cancer but also leads to adverse health outcomes including metabolic syndrome, cardiovascular diseases, and increased mortality, thereby constituting a significant social burden for women (4, 21, 22). Obesity is a recognized global epidemic, affecting an estimated 800 million people worldwide, with obesity rates particularly high in the United States, where it accounts for about 42 percent of American adults (23). Obesity rates among women of reproductive age in the United States are also increasing: in 2002, about 23% of women of reproductive age were classified as obese, and by 2021, more than 50 percent of women of reproductive age are overweight or obese, which has become one of the leading risk factors for infertility (24, 25).

Obesity is largely considered to impair ovulatory function primarily through alterations in the hypothalamic-pituitary-ovarian (HPO) axis, affecting hormone release and leading to ovulatory dysfunction (26, 27). Excessive fat deposition exacerbates this condition, significantly reducing conception rates among obese women (28). Additionally, factors such as poor oocyte maturation quality result in decreased embryonic potential and increased difficulties in achieving pregnancy in obese women without ovulatory dysfunction (19, 20, 29). Even with assisted reproductive technologies like *in vitro* fertilization (IVF), the success rates remain lower in obese women compared to those with normal weight (30).

Obesity is widely acknowledged as a pivotal factor contributing to infertility, prompting extensive investigation into the role of obesity-related indicators in infertility screening. This study analyzed NHANES data to assess the efficacy of five indices, BRI, RFM, BMI, WC, and LAP, in distinguishing infertility cases among U.S. adults. ROC curve analysis identified BRI as the most predictive indicator. Furthermore, our findings reveal that the association between BRI and infertility is more robust within a specific range compared to traditional obesity markers such as BMI and WC. Although BMI is a globally recognized standard for obesity assessment, it often overlooks the distribution of body fat and muscle, particularly the influence of varying visceral fat content on physiological health (31). This limitation underscores the differential impact of visceral fat on overall well-being. Our study demonstrates that with each unit increase in BRI, the risk of infertility escalates by 12%.

Maternal overweight and obesity before conception have been independently associated with an elevated risk of neurodevelopmental disorders in offspring, including attention-deficit/hyperactivity disorder, autism spectrum disorder, conduct disorder (32), and psychosis, as well as pregnancy complications including gestational diabetes, preeclampsia, preterm birth, and cesarean delivery (33). These findings highlight the intergenerational effects of maternal obesity and underscore the need for early preventive strategies. Mechanistically, chronic inflammation and oxidative stress associated with obesity may impair key reproductive processes, including hormone production and ovulation (29). Preconception lifestyle interventions and weight loss have been shown to restore menstrual regularity, improve ovulation, and significantly enhance fertility and pregnancy outcomes in obese women (34–36). The incorporation of obesity-related indicators, such as BRI and RFM, into preconception evaluations may enhance early risk stratification and facilitate targeted lifestyle interventions to improve reproductive outcomes.

The BRI was initially proposed in 2013 as a novel obesity metric, and since then, several studies have confirmed its association with various systemic diseases (11). For instance, Gao et al. found a positive correlation between BRI and colorectal cancer incidence in a cohort of 53,766 participants, with BRI exhibiting superior predictive capability compared to BMI and other anthropometric measures (12). Similarly, Li et al., in a study of 47,303 participants, found BRI to be a moderate yet independent risk factor for metabolic syndrome (16). Furthermore, Qiu et al. observed a nonlinear association between elevated BRI and the prevalence of diabetes and prediabetes among U.S. adults, confirming that BRI outperforms BMI and waist circumference as an obesity-related marker (37). Zhang et al. also suggested that BRI could be a valuable predictor of depression (14).

Subgroup analysis revealed a significant trend: individuals aged 35 and above are more likely to report infertility, indicating that age plays an independent role in the pathogenesis of infertility. Additionally, the findings demonstrate that health issues such as smoking, diabetes, and hypertension further exacerbate the risk of infertility, highlighting the negative impact of unhealthy lifestyle habits and chronic diseases on reproductive health. Also, alcohol consumption was also identified as a potential risk factor (*p* = 0.053), suggesting that even moderate alcohol intake may adversely affect fertility. These findings underscore the importance of a comprehensive assessment of lifestyle and health status for screening and preventing infertility.

This study has several strengths. The large sample size enhances the statistical power and reliability of the findings. Additionally, the study appropriately adjusted for numerous potential confounding variables, thereby improving the accuracy of the association between risk factors and infertility prevalence. Furthermore, a nonlinear relationship was observed between the BRI and infertility risk, providing additional support for the existence of threshold phenomena. However, there are also limitations to consider. A primary limitation is the retrospective cross-sectional design of the study, which precludes the establishment of causality. Infertility was identified through interviews, which may be subject to recall bias or misclassification. Moreover, the NHANES database lacks information on the duration of infertility, frequency and timing of intercourse, semen quality, diagnostic details of all infertility causes, and polycystic ovary syndrome (PCOS). These limitations may restrict further exploration of underlying mechanisms. Additionally, the study focused exclusively on the U.S. population, making it unclear whether the findings are generalizable to other countries. Finally, we were unable to account for participants’ medication use, including types, frequencies, and durations, which may influence infertility risk.

## 5 Conclusion

Our findings demonstrate that five screening indicators, RFM, BRI, BMI, WC, and LAP, are positively associated with infertility risk. BRI may serve as a supplementary tool for early risk assessment in screening. Individuals aged >35 years, smokers, and those with diabetes or hypertension need particular attention. Clinicians can leverage BRI to identify individuals at elevated risk of infertility, thereby improving screening efficacy, optimizing preconception care, and enhancing conception outcomes.

## Data Availability

Publicly available datasets were analyzed in this study. This data can be found here: This study examined datasets that were accessible to the public. The location of this data can be accessed here: http://www.cdc.gov/nhanes.

## References

[1] Pisarska MD Chan JL Lawrenson K Gonzalez TL Wang ET. Genetics and epigenetics of infertility and treatments on outcomes. J Clin Endocrinol Metab. (2019) 104:1871–86. doi: 10.1210/jc.2018-01869, PMID: 30561694 PMC6463256

[2] Peterson BD Sejbaek CS Pirritano M Schmidt L. Are severe depressive symptoms associated with infertility-related distress in individuals and their partners? Hum Reprod. (2014) 29:76–82. doi: 10.1093/humrep/det412, PMID: 24256990

[3] Boivin J Bunting L Collins JA Nygren KG. International estimates of infertility prevalence and treatment-seeking: potential need and demand for infertility medical care. Hum Reprod. (2007) 22:1506–12. doi: 10.1093/humrep/dem046, PMID: 17376819

[4] Carson SA Kallen AN. Diagnosis and management of Infertility: a review. JAMA. (2021) 326:65–76. doi: 10.1001/jama.2021.4788, PMID: 34228062 PMC9302705

[5] Inhorn MC Patrizio P. Infertility around the globe: new thinking on gender, reproductive technologies and global movements in the 21st century. Hum Reprod Update. (2015) 21:411–26. doi: 10.1093/humupd/dmv016, PMID: 25801630

[6] Poston L Caleyachetty R Cnattingius S Corvalán C Uauy R Herring S . Preconceptional and maternal obesity: epidemiology and health consequences. Lancet Diabetes Endocrinol. (2016) 4:1025–36. doi: 10.1016/S2213-8587(16)30217-0, PMID: 27743975

[7] Tang J Xu Y Wang Z Ji X Qiu Q Mai Z . Association between metabolic healthy obesity and female infertility: the national health and nutrition examination survey, 2013–2020. BMC Public Health. (2023) 23:1524. doi: 10.1186/s12889-023-16397-x, PMID: 37563562 PMC10416469

[8] Wang D Chen Z Wu Y Ren J Shen D Hu G . Association between two novel anthropometric measures and type 2 diabetes in a Chinese population. Diabetes Obes Metab. (2024) 26:3238–47. doi: 10.1111/dom.15651, PMID: 38783824

[9] Kahn HS. The "lipid accumulation product" performs better than the body mass index for recognizing cardiovascular risk: a population-based comparison. BMC Cardiovasc Disord. (2005) 5:26. doi: 10.1186/1471-2261-5-26, PMID: 16150143 PMC1236917

[10] Zhang X Ma N Lin Q Chen K Zheng F Wu J . Body roundness index and all-cause mortality among US adults. JAMA Netw Open. (2024) 7:e2415051. doi: 10.1001/jamanetworkopen.2024.15051, PMID: 38837158 PMC11154161

[11] Thomas DM Bredlau C Bosy-Westphal A Mueller M Shen W Gallagher D . Relationships between body roundness with body fat and visceral adipose tissue emerging from a new geometrical model. Obesity (Silver Spring). (2013) 21:2264–71. doi: 10.1002/oby.20408, PMID: 23519954 PMC3692604

[12] Gao W Jin L Li D Zhang Y Zhao W Zhao Y . The association between the body roundness index and the risk of colorectal cancer: a cross-sectional study. Lipids Health Dis. (2023) 22:53. doi: 10.1186/s12944-023-01814-2, PMID: 37072848 PMC10111650

[13] Wei C Zhang G. Association between body roundness index (BRI) and gallstones: results of the 2017–2020 national health and nutrition examination survey (NHANES). BMC Gastroenterol. (2024) 24:192. doi: 10.1186/s12876-024-03280-1, PMID: 38840060 PMC11155175

[14] Zhang L Yin J Sun H Dong W Liu Z Yang J . The relationship between body roundness index and depression: a cross-sectional study using data from the National Health and Nutrition Examination Survey (NHANES) 2011–2018. J Affect Disord. (2024) 361:17–23. doi: 10.1016/j.jad.2024.05.153, PMID: 38815765

[15] Zhang G Zhang H Fu J Zhao Y. Atherogenic index of plasma as a mediator in the association between body roundness index and depression: insights from NHANES 2005–2018. Lipids Health Dis. (2024) 23:183. doi: 10.1186/s12944-024-02177-y, PMID: 38867232 PMC11167922

[16] Li Z Fan C Huang J Chen Z Yu X Qian J. Non-linear relationship between the body roundness index and metabolic syndrome: data from National Health and Nutrition Examination Survey (NHANES) 1999–2018. Br J Nutr. (2024) 131:1852–9. doi: 10.1017/S0007114524000357, PMID: 38356387

[17] Deng C Ke X Lin L Fan Y Li C. Association between indicators of visceral lipid accumulation and infertility: a cross-sectional study based on U.S. women. Lipids Health Dis. (2024) 23:186. doi: 10.1186/s12944-024-02178-x, PMID: 38872138 PMC11170861

[18] Liu D Luo X Zhou K. Association between current relative fat mass and history of female infertility based on the NHANES survey. Sci Rep. (2025) 15:6294. doi: 10.1038/s41598-025-89417-y, PMID: 39984538 PMC11845496

[19] Hou YJ Zhu CC Duan X Liu HL Wang Q Sun SC. Both diet and gene mutation induced obesity affect oocyte quality in mice. Sci Rep. (2016) 6:18858. doi: 10.1038/srep18858 26732298 PMC4702149

[20] Metwally M Cutting R Tipton A Skull J Ledger WL Li TC. Effect of increased body mass index on oocyte and embryo quality in IVF patients. Reprod Biomed Online. (2007) 15:532–8. doi: 10.1016/S1472-6483(10)60385-9, PMID: 18044034

[21] Rizzuto I Behrens RF Smith LA. Risk of ovarian cancer in women treated with ovarian stimulating drugs for infertility. Cochrane Database Syst Rev. (2019) 6:CD008215. doi: 10.1002/14651858.CD008215.pub3, PMID: 31207666 PMC6579663

[22] Gleason JL Shenassa ED Thoma ME. Self-reported infertility, metabolic dysfunction, and cardiovascular events: a cross-sectional analysis among U.S. women. Fertil Steril. (2019) 111:138–46. doi: 10.1016/j.fertnstert.2018.10.009, PMID: 30458992

[23] Campbell LA Kombathula R Jackson CD. Obesity in adults. JAMA. (2024) 332:600. doi: 10.1001/jama.2024.5126, PMID: 39102238

[24] Vahratian A. Prevalence of overweight and obesity among women of childbearing age: results from the 2002 National Survey of Family Growth. Matern Child Health J. (2009) 13:268–73. doi: 10.1007/s10995-008-0340-6, PMID: 18415671 PMC2635913

[25] Paredes C Hsu RC Tong A Johnson JR. Obesity and pregnancy. NeoReviews. (2021) 22:e78–87. doi: 10.1542/neo.22-2-e78, PMID: 33526637

[26] Jain A Polotsky AJ Rochester D Berga SL Loucks T Zeitlian G . Pulsatile luteinizing hormone amplitude and progesterone metabolite excretion are reduced in obese women. J Clin Endocrinol Metab. (2007) 92:2468–73. doi: 10.1210/jc.2006-2274, PMID: 17440019

[27] Broughton DE Moley KH. Obesity and female infertility: potential mediators of obesity's impact. Fertil Steril. (2017) 107:840–7. doi: 10.1016/j.fertnstert.2017.01.017, PMID: 28292619

[28] Jungheim ES Moley KH. Current knowledge of obesity's effects in the pre-and periconceptional periods and avenues for future research. Am J Obstet Gynecol. (2010) 203:525–30. doi: 10.1016/j.ajog.2010.06.043, PMID: 20739012 PMC3718032

[29] Snider AP Wood JR. Obesity induces ovarian inflammation and reduces oocyte quality. Reproduction. (2019) 158:R79–90. doi: 10.1530/REP-18-0583, PMID: 30999278

[30] Shah DK Missmer SA Berry KF Racowsky C Ginsburg ES. Effect of obesity on oocyte and embryo quality in women undergoing in vitro fertilization. Obstet Gynecol. (2011) 118:63–70. doi: 10.1097/AOG.0b013e31821fd360, PMID: 21691164

[31] Rothman KJ. BMI-related errors in the measurement of obesity. Int J Obes. (2008) 32:S56–9. doi: 10.1038/ijo.2008.87, PMID: 18695655

[32] Duko B Mengistu TS Stacey D Moran LJ Tessema G Pereira G . Associations between maternal preconception and pregnancy adiposity and neuropsychiatric and behavioral outcomes in the offspring: a systematic review and meta-analysis. Psychiatry Res. (2024) 342:116149. doi: 10.1016/j.psychres.2024.116149, PMID: 39278191

[33] Prodan NC Schmidt M Hoopmann M Abele H Kagan KO. Obesity in prenatal medicine: a game changer? Arch Gynecol Obstet. (2024) 309:961–74. doi: 10.1007/s00404-023-07251-x, PMID: 37861742 PMC10867045

[34] Legro RS Hansen KR Diamond MP Steiner AZ Coutifaris C Cedars MI . Effects of preconception lifestyle intervention in infertile women with obesity: the FIT-PLESE randomized controlled trial. PLoS Med. (2022) 19:e1003883. doi: 10.1371/journal.pmed.1003883, PMID: 35041662 PMC8765626

[35] Mohammadian F Delavar MA Behmanesh F Azizi A Esmaeilzadeh S. The impact of health coaching on the prevention of gestational diabetes in overweight/obese pregnant women: a quasi-experimental study. BMC Womens Health. (2023) 23:619. doi: 10.1186/s12905-023-02750-0, PMID: 37990232 PMC10664614

[36] Silvestris E de Pergola G Rosania R Loverro G. Obesity as disruptor of the female fertility. Reprod Biol Endocrinol. (2018) 16:22. doi: 10.1186/s12958-018-0336-z, PMID: 29523133 PMC5845358

[37] Qiu L Xiao Z Fan B Li L Sun G. Association of body roundness index with diabetes and prediabetes in US adults from NHANES 2007–2018: a cross-sectional study. Lipids Health Dis. (2024) 23:252. doi: 10.1186/s12944-024-02238-2, PMID: 39154165 PMC11330595

