# Correction: Comparative analysis of five obesity-related indicators for predicting infertility in U.S. adults

**DOI:** 10.3389/fnut.2026.1902250

**Published:** 2026-06-23

**Authors:** Ke-Qin Yu, Wei-Qiang Zhao, Ting Zhang

**Affiliations:** 1 Department of Gynaecology & Obstetrics, The First Affiliated Hospital of Zhejiang Chinese Medical University (Zhejiang Provincial Hospital of Chinese Medicine), Hangzhou, China; 2 The First Clinical College of Zhejiang Chinese Medical University, Hangzhou, China; 3 Department of Orthopaedic & Traumatology, The First Affiliated Hospital of Zhejiang Chinese Medical University (Zhejiang Provincial Hospital of Chinese Medicine), Hangzhou, China

**Keywords:** body roundness index, obesity-index, relative fat mass, lipid accumulation product, National Health and Nutrition Examination Survey

Affiliation 1 and 3 were incorrectly written as, “Department of Gynaecology & Obstetrics, The First Affiliated Hospital of Zhejiang Chinese Medical University (Zhejiang Provincial Hospital of Traditional Chinese Medicine), Hangzhou, China” and “Department of Orthopaedic & Traumatology, The First Affiliated Hospital of Zhejiang Chinese Medical University (Zhejiang Provincial Hospital of Traditional Chinese Medicine), Hangzhou, China.” The correct affiliations should read as, “Department of Gynaecology & Obstetrics, The First Affiliated Hospital of Zhejiang Chinese Medical University (Zhejiang Provincial Hospital of Chinese Medicine), Hangzhou, China” and “Department of Orthopaedic & Traumatology, The First Affiliated Hospital of Zhejiang Chinese Medical University (Zhejiang Provincial Hospital of Chinese Medicine), Hangzhou, China.”

The author, Wei-Qiang Zhao, was erroneously assigned to affiliation (Department of Gynaecology & Obstetrics, The First Affiliated Hospital of Zhejiang Chinese Medical University (Zhejiang Provincial Hospital of Traditional Chinese Medicine), Hangzhou, China). The correct affiliation for the author, Wei-Qiang Zhao, should be assigned to “The First Clinical College of Zhejiang Chinese Medical University, Hangzhou, China and “Department of Orthopaedic & Traumatology, The First Affiliated Hospital of Zhejiang Chinese Medical University (Zhejiang Provincial Hospital of Chinese Medicine), Hangzhou, China”.

An incorrect **Funding** statement was provided. The incorrect **Funding** statement read as,

“The author(s) declare that financial support was received for the research and/or publication of this article. This study was funded by the Natural Science Foundation of Zhejiang Province (No. BY24H040049) and the Zhejiang Province Traditional Chinese Medicine Science and Technology Plan Project (2019ZB044).”

The correct **Funding** statement should read as,

“The author(s) declared that financial support was received for this work and/or its publication. This study was funded by the Joint Funds of the Zhejiang Provincial Natural Science Foundation of China (Grant No. LBY24H040008) and the Zhejiang Province Traditional Chinese Medicine Science and Technology Plan Project (2019ZB044).”

The original version of this article has been updated.

